# How Gene Therapy Research Has Evolved and the Future of Oversight

**DOI:** 10.1017/jme.2025.10211

**Published:** 2026

**Authors:** Alexandra Finch, Lawrence O. Gostin

**Affiliations:** 1 O’Neill Institute for National and Global Health Law, Georgetown University Law Center, United States; 2 Georgetown University, United States

**Keywords:** gene therapy research, gene editing, oversight, Recombinant DNA Advisory Committee, Novel and Exceptional Technology and Research Advisory Committee (NExTRAC)

## Abstract

Commenting on Cargill’s article, this Commentary examines how gene therapy research is regulated in the United States and how oversight of the field has developed. It discusses recent applications of gene therapy technologies and their implications for oversight, and of the impact of ordered cuts to NIH-funded research on gene therapy developments more broadly. Ultimately, it underscores the need for adaptive oversight frameworks for research involving emerging biotechnologies that balance scientific innovation, safety, and ethical considerations, and for effective public engagement on the acceptable use of these technologies, notwithstanding the discontinuation of NIH’s advisory mechanism established for this purpose.

Cargill’s article critiques the 2019 amendments to the NIH Guidelines for Research Involving Recombinant DNA Molecules (NIH Guidelines), which streamlined oversight of gene therapy research in the United States, including by eliminating public review of gene therapy protocols by the Recombinant DNA Advisory Committee (RAC). Cargill proficiently traces the history of regulatory oversight in the field, arguing that the changes leave important social and ethical concerns involving emerging biotechnologies inadequately addressed, using the test case of xenotransplantation.

One of us (LOG) chaired the Institute of Medicine (IOM) Committee on the Independent Review and Assessment of the Activities of RAC,^1^ tasked with assessing the need for RAC review of gene therapy protocols in 2013. Since then, gene therapy research has made substantial progress, marked by advances in science, clinical applications, and the development of FDA-approved therapies. Commenting on Cargill’s article, we examine how gene therapy research is regulated today and how oversight has changed since the IOM Committee, then highlight recent applications of gene therapy technologies and their implications for oversight.

Regulating gene therapy research ultimately requires a delicate balance between safety and speed, scientific innovation and ethics.^2^ Scientific oversight helps ensure that the clinical benefits outweigh risks to patients, and lay or ethical oversight provides an important assurance that societal values are respected, each enabling public trust. Federal oversight should continue to adapt to reflect advances in science and their associated risks, including for society, and Cargill presents some useful recommendations for that adaptation.

## How Gene Therapy Research is Regulated Today

Today, FDA primarily regulates human gene therapy *products.* Gene therapy researchers must submit an investigational new drug (IND) application to FDA for authorization before they can begin clinical tests in humans.^3^ Once an IND is authorized, gene therapy protocols are reviewed at the institutional level by an Institutional Review Board (IRB) to ensure compliance with regulations to protect human research subjects in clinical investigations,^4^ and by an Institutional Biosafety Committee (IBC) at each research site for compliance with the NIH Guidelines.^5^ Compliance with the NIH Guidelines is mandatory for NIH-funded research.^6^ Primary regulatory authority lies with the FDA to ensure that only sufficiently safe and effective therapies reach the market, and with IRBs and IBCs to ensure trial safety.

Meeting as needed — approximately biannually — the Novel and Exceptional Technology and Research Advisory Committee (NExTRAC), comprised of scientists, ethicists, and patient and public representatives, advised NIH on the conduct and oversight of research involving emerging biotechnologies and served as a public forum for deliberating the scientific, safety, and ethical issues related to their use,^7^ rather than serving as an additional regulatory step for any given therapy.

## History of Gene Therapy Research Oversight

When gene therapy research emerged, it marked a significant milestone in biomedical research but raised concerns among the scientific community, leading NIH to establish RAC in 1974. RAC’s mission was to advise the NIH director on emerging biotechnologies and provide a public forum for addressing ethical and scientific issues associated with their use, later expanding to reviewing gene therapy protocols.^8^ RAC reviewed its first protocol in 1989 and was instrumental in shaping early gene therapy research oversight. But in 1995, NIH and FDA agreed to jointly determine which protocols warranted RAC review.^9^ Safety concerns rematerialized in 1999 with a patient death during a gene therapy trial, prompting greater scrutiny.

After a decade of scientific advances, NIH requested IOM to assess the state of gene transfer science and the need for RAC review of individual protocols. The IOM Committee found that while scientific, social, and ethical questions would continue to be raised, not all gene transfer research was considered novel and therefore not all protocols would warrant public review.^10^ It deemed existing regulatory frameworks sufficient to ensure its safe and ethical conduct and recommended that RAC’s role be limited to reviewing truly novel protocols.

In 2016, NIH accordingly revised the NIH Guidelines, empowering IRBs and IBCs to determine whether RAC review was warranted based on three criteria: if the protocol presented an unknown risk; if it relied on preclinical data obtained using a model of unknown value; or if the delivery method was associated with little-known toxicities.^11^ RAC review was limited to cases meeting those criteria or those determined by the NIH director to present significant scientific, societal, or ethical concerns.^12^ During this period, only a fraction (approximately 1%) of relevant protocols required RAC review.^13^ By 2018, NIH proposed additional revisions to streamline oversight of gene therapy research.^14^ NIH and FDA leaders envisioned “using RAC as an advisory board” on emerging biotechnologies.^15^ In 2019, NIH stopped registering new human gene transfer protocols and stopped convening RAC to review them,^16^ refocusing the NIH Guidelines on biosafety and leaving primary clinical oversight to the FDA. That year, NExTRAC was established and thereafter focused on the implications of gene editing technologies, neurotechnologies, synthetic biology, and gene drives, the last of which led to recent revisions to the NIH Guidelines.^17^ Fifty years on from RAC, gene therapy research is no longer novel, though its applications continue to evolve.

However, in May 2025, reflecting a pattern of eroding scientific expertise across the federal government, NExTRAC members were informed that their panel was being sunset as part of broader federal efforts to improve efficiency.^18^ At NExTRAC’s last meeting on September 29, 2025, the NIH director noted that despite the panel’s sunsetting, public discussion on emerging biotechnologies would continue through “alternative avenues,”^19^ though it was not immediately clear what those avenues were. Meanwhile, FDA leadership revised regulatory requirements to facilitate more efficient clinical translation of gene therapy research.^20^

## New Gene Therapy Applications

The years following the IOM Committee brought significant advances in gene therapy. FDA’s approval of a recombinant DNA product for melanoma in 2016 was a key milestone.^21^ As of December 2025, FDA had approved dozens of cell and gene therapy products,^22^ and the agency previously predicted it would approve 10 to 20 gene therapies annually starting in 2025.^23^ Innovations like CAR-T cell therapies have demonstrated efficacy against cancers,^24^ leading to the approval of cell-based gene therapies for cancer. And new digital technologies like artificial intelligence (AI) hold immense potential for helping us understand and diagnose conditions, but introduce distinct risks including privacy infringement,^25^ and concerns associated with their integration into existing biotechnologies, for example with AI-enabled gene editing.

The development of gene editing technologies like CRISPR has expanded the possibilities for treating genetic diseases previously considered untreatable. CRISPR-Cas9’s ability to make precise modifications to DNA makes it a powerful gene editing tool. In 2016, RAC publicly reviewed the first protocol for a Phase I trial using CRISPR-edited T cells.^26^ Since then, CRISPR has been clinically applied to sickle-cell disease, liver disorders, congenital eye disease, HIV, and cancer. The technology also holds promise for microbiome editing and developing viral vaccines.

Human gene editing in clinical practice is currently limited to somatic (non-heritable) applications. The technology still raises safety concerns, including risks of off-target effects or immune responses, and ethical challenges defining its acceptable use. But these challenges are not new. Heritable human gene editing, however, has been the subject of intense debate,^27^ and there is inadequate preclinical evidence of its efficacy.^28^ NIH does not fund its use in human embryos, Congress has prohibited FDA review of that research,^29^ and WHO’s Director-General has said it would be “irresponsible” to proceed with its clinical application.^30^

Instead, the gene editing mechanism that recently concerned NIH’s NExTRAC is gene drives — processes capable of propagating select traits through generations of an organism,^31^ like sterility or resistance to disease. Gene drives using CRISPR-Cas9 technology could reduce the population of mosquitos carrying malaria,^32^ a disease responsible for almost 600,000 annual deaths, mostly in children under five.^33^ Still, gene drives have uncertain risks and raise ethical and ecological concerns, underscoring the need for oversight.

Cargill argues that the oversight framework is insufficient to address ethical issues with emerging biotechnologies. A central contention is that NExTRAC inadequately fulfilled its “horizon scanning” role, evidenced by its lack of engagement with xenotransplantation despite her assessment that it met NExTRAC’s criteria for review. At its inaugural meeting, NExTRAC identified xenotransplantation as a technology that it could debate.^34^ But when NExTRAC developed its framework for determining if a biotechnology warranted public deliberation, it acknowledged the framework’s scope would capture more biotechnologies than it could ultimately address and that the prompts were not a checklist.^35^ If NExTRAC had not been slated to sunset, it might have continued to address applications of xenotransplantation among other emerging biotechnologies. However, its structure and limited resources, as Cargill identifies, prevented it from doing the kind of iterative deliberation that could cover the growing number, scope, and complexity of emerging biotechnologies. Any new body – an alternate avenue – need not be so limited. However, securing the additional financing required to support such thorough oversight appears unlikely at present (as discussed below).

Another of Cargill’s contentions is that NExTRAC did not support institutional IRBs and IBCs with specialized expertise on emerging biotechnologies — a role RAC fulfilled. IRBs and IBCs have limited capacity to address ethical issues. The FDA has issued guidance on xenotransplantation,^36^ but is not empowered to consider values-based concerns. Standing national review, as Cargill recommends, would alleviate the burden on IRBs and IBCs for consulting experts on social and ethical risks associated with xenotransplantation,^37^ as with other emerging biotechnologies, while while achieving broader, more inclusive dialogue and consensus.

## Impact of Cuts to NIH-Funded Research

In 2025, the future of gene therapy research in the United States became uncertain as the Trump administration sharply reduced federal funding for scientific research, including gene therapy. First, in February, the administration issued guidance reducing and capping previously negotiated indirect cost rates on existing and future NIH grants.^38^ Indirect costs cover essential outlays in gene therapy and other research such as laboratory space and research equipment. Second, as of November, the administration had frozen or terminated approximately $2.3 billion in unspent funds across nearly 2,500 NIH grants, including 120 genetics and genomics grants.^39^ Legal challenges to NIH’s grant terminations have led to partial reinstatements, but many awards remain frozen or under review.^40^

Further disruptions could impact the continuation of NIH-funded research on ultra-rare neurological diseases,^41^ for developing CRISPR-based gene therapies for brain diseases,^42^ and for developing tools for safe somatic gene editing.^43^ Any further retrenchment on NIH funding for gene therapy research could slow progress on potentially life-saving new therapies.

## Conclusion

Over the past half century, gene therapy research has evolved from an uncertain science to an established field, as has its oversight structure. Public engagement on ethical issues concerning emerging biotechnologies remains important as it promotes transparency and builds public trust in science. Public deliberations also enable marginalized perspectives to be effectively incorporated into decision-making about the acceptable uses of emerging biotechnologies. Public forums like NExTRAC play a crucial role in public engagement and, as advisory bodies, are unlikely to introduce inefficiencies into the regulatory process. New biotechnologies will continue to emerge and carry broad societal implications. Whether through the reinstatement of a reformed NExTRAC or some alternative body comprised of scientific experts and diverse other stakeholders (government, legal, ethical, and lay), oversight and public deliberation of the ethical and societal implications of emerging biotechnologies should continue in the public interest. And any such body would serve its public purpose better if it were adequately financed and its deliberations occurred on an ongoing basis.^44^ As gene therapy research continues to push the limits of science, the oversight framework must keep pace and evolve with it for its responsible use.
